# Supplementary material for: Preliminary Investigation of Side Effects of Polymyxin B Administration in Hospitalized Horses
Source: Antibiotics (Basel). 2023 May 5;12(5):854. doi: 10.3390/antibiotics12050854 (PMC10215903; doi:10.3390/antibiotics12050854)
Supplement: Supplementary file 1 [file antibiotics-12-00854-s001.zip › Supplementary 1_Neurological exam.pdf]

Universität Zürich UZH

Equine Department
Clinics of Equine Internal Medicine

Polymyxin study - Video analysis sheet
Evaluation of neurological examination

Evaluator: _____
Randomization number: _____

Description and analysis of neurological examination:

____
____
____

**Evaluation of gait at walk**	□ no deficits	□ slight deficits	□ obvious deficits		□ not assessable
**Circling**	□ no deficits	□ slight deficits	□ obvious deficits	□ circumduction	□ not assessable
**Tail-pull**	□ no deficits	□ slight deficits	□ obvious deficits	□ weakness	□ not assessable
**Elevated head**	□ no deficits	□ slight deficits	□ obvious deficits		□ not assessable
**Backing**	□ no deficits	□ slight deficits	□ obvious deficits		□ not assessable
**Walk over obstacle**	□ no deficits	□ slight deficits	□ obvious deficits	□ stumbling	□ not assessable
**Proprioception**	□ no deficits			□ ≥2 legs with reduced proprioception	□ not assessable
**Standing sway test**	□ no deficits	□ slight deficits	□ obvious deficits	□ weakness	□ not assessable
**Spinal reflexes (anal, cutaneous trunci)**	□ no deficits			□ reduced<br>□ increased	□ not assessable
**Cutaneous sensation**	□ no deficits			□ reduced<br>□ increased	□ not assessable
**Cranial nerves**	□ no deficits	Deficits: ______________________________			□ not assessable
**Weakness**	□ yes, present			□ no, absent	□ not assessable

Ataxia grading (0-5/5): _____

Grad 0: normal
Grad 1: deficits barely perceptible – worsened with provocation
Grad 2: deficits noted at walk
Grad 3: deficits noted at rest and walk, nearly falls with provocation
Grad 4: falls or nearly falls at normal gait
Grad 5: recumbent
