# Supplementary material for: Preliminary Investigation of Side Effects of Polymyxin B Administration in Hospitalized Horses
Source: Antibiotics (Basel). 2023 May 5;12(5):854. doi: 10.3390/antibiotics12050854 (PMC10215903; doi:10.3390/antibiotics12050854)
Supplement: Supplementary file 1 [file antibiotics-12-00854-s001.zip › Supplementary 2_ Blood analyses.pdf]

Supplementary 2

Hematology and clinical chemistry results of 20 hospitalized horses receiving PolyB treatment (6000IU/kg IV q12h) at the first day of treatment.

**Parameter (unit)**	**Hematocrit (%)**	**Hemoglobin (g/dL)**	**Erythrocytes (10E6/µL)**	**Leukocytes (10E3/µL)**	**Thrombocytes (10E3/µL)**	**Bands (10E3/µL)**	**Segmented Neutrophils (10E3/µL)**	**Monocytes (10E3/µL)**	**Eosinophils (10E3/µL)**	**Lymphocytes (10E3/µL)**
**Reference range**	**30-42**	**10.8-14.9**	**6.2-9.0**	**4.7-8.2**	**119-250**	**<0.1**	**3.0-5.8**	**<0.4**	**<0.1**	**1.0-3.5**
Horse A	-/-	-/-	-/-	4.2	-/-	-/-	-/-	-/-	-/-	-/-
Horse B	16	6	-/-	2.7	114	-/-	-/-	-/-	-/-	-/-
Horse C	34	12	7	2.8	243	0.5	0.6	0.0	0.0	1.8
Horse D	28	10	6	5.2	171	0.2	4.3	0.1	0.0	0.6
Horse E		-/-	-/-	8.7	-/-	-/-	-/-	-/-	-/-	-/-
Horse F	31	12	7	5.0	213	1.2	2.5	0.1	0.0	1.2
Horse G	27	10	6	3.7	75	0.0	3.0	0.2	0.0	0.5
Horse H	-/-	-/-	-/-	-/-	-/-	-/-	-/-	-/-	-/-	-/-
Horse I	32	12	7	4.5	164	0.5	2.9	0.3	0.0	0.7
Horse J	-/-	-/-	-/-	-/-	-/-	3.9	-/-	-/-	-/-	-/-
Horse K	-/-	-/-	-/-	3.7	-/-	-/-	-/-	-/-	-/-	-/-
Horse L	-/-	-/-	-/-	3.7	-/-	-/-	-/-	-/-	-/-	-/-
Horse M	26	9	5	4.0	69	0.1	2.5	0.1	0.0	1.2
Horse N	30	11	7	8.9	87	0.4	7.9	0.1	0.0	0.4
Horse O	40	15	10	8.2	215	1.8	5.2	0.0	0.0	1.2
Horse P	29	11	6	2.2	67	0.0	0.8	0.0	0.0	1.3
Horse Q	40	15	8	3.3	169	0.8	0.9	0.1	0.0	1.4
Horse R	30	-/-	-/-	3.7	-/-	0.2	1.1	0.2	0.0	2.2
Horse S	-/-	-/-	-/-	5.9	-/-	-/-	-/-	-/-	-/-	-/-
Horse T	43	16	8	2.2	181	0.3	0.3	0.1	0.0	1.5

Parameter (unit)	Bilirubin (µmol/L)	Urea (mmol/L)	Creatinine (µmol/L)	SDMA (µg/dL)	Protein (g/L)	Albumin (g/L)	AP (U/L)	ASAT (U/L)	GGT (U/L)	GLDH (U/L)	SDH (U/L)	LDH (U/L)	CK (U/L)
**Reference range**	**17.4-35.2**	**3.5-7.0**	**82-147**	**<15**	**25-70**	**25-34**	**81-183**	**229-393**	**6-31**	**0.5-2.2**	**0.1-7.6**	**369-822**	**112-305**
Horse A	-/-	5.4	90	12	-/-	-/-	-/-	-/-	-/-	-/-	-/-	-/-	-/-
Horse B	-/-	2.5	83	10	-/-	-/-	-/-	-/-	-/-	-/-	-/-	-/-	-/-
Horse C	35	4.1	104	7	44	24	100	319	9	32	16	1158	1025
Horse D	-/-	3.0	109	10	55	29	-/-	-/-	-/-	-/-	-/-	-/-	-/-
Horse E	-/-	-/-	-/-	9	-/-	-/-	-/-	-/-	-/-	-/-	-/-	-/-	-/-
Horse F	40	4.0	92	6	52	28	148	437	22	45	11	1629	3473
Horse G	63	1.7	99	-/-	56	28	94	340	15	6	8	759	557
Horse H	-/-	-/-	-/-	10	-/-	-/-	-/-	-/-	-/-	-/-	-/-	-/-	-/-
Horse I	82	4.0	89	12	56	23	246	297	8	4	5	923	460
Horse J	-/-	-/-	-/-	11	-/-	-/-	-/-	-/-	-/-	-/-	-/-	-/-	-/-
Horse K	-/-	4.6	102	10	-/-	-/-	-/-	-/-	-/-	-/-	-/-	-/-	-/-
Horse L	-/-	3.4	88	10	-/-	17	-/-	-/-	-/-	-/-	-/-	-/-	-/-
Horse M	108	2.8	97	10	53	28	153	598	10	6	2	963	1112
Horse N	117	6.0	146	18	52	22	178	714	14	16	12	1167	1282
Horse O	72	4.1	91	9	41	21	98	425	10	10	12	1184	3614
Horse P	11	2.1	104	7	61	28	177	448	15	11	17	970	-/-
Horse Q	114	2.6	104	12	48	24	263	531	18	8	124	1129	1075
Horse R	-/-	-/-	-/-	11	63	-/-	-/-	-/-	-/-	-/-	-/-	-/-	-/-
Horse S	63	7.0	176	19	62	27	717	678	98	29	87	1931	137
Horse T	56	4.9	117	11	42	22	146	293	11	6	4	880	1471

Parameter (unit)	Sodium (mmol/L)	Potassium (mmol/L)	Chlorid (mmol/L)	Calcium (mmol/L)	Magnesium (mmol/L)	Phosphate (mmol/L)	Glucose (mmol/L)	SAA (mg/L)	Fibrinogen (g/L)
**Reference range**	**133-141**	**2.3-4.6**	**98-106**	**2.9-3.3**	**0.6-0.8**	**0.7-1.3**	**4.5-5.9**	**<1.2**	**1.3-2.9**
Horse A	-/-	-/-	-/-	-/-	-/-	-/-	-/-	2148	4.0
Horse B	-/-	-/-	-/-	-/-	-/-	-/-	-/-	8745	6.2
Horse C	129.0	3.1	95.0	2.4	0.31	0.6	14	106	1.8
Horse D	135.0	3.6	99.0	2.6	-/-	-/-	7	-/-	-/-
Horse E	-/-	-/-	-/-	-/-	-/-	-/-	-/-	-/-	-/-
Horse F	-/-	2.9	92.0	2.7	0.55	0.7	9	369	2.3
Horse G	138.0	3.6	105.0	2.7	0.59	0.6	6	1852	2.7
Horse H	-/-	-/-	-/-	-/-	-/-	-/-	-/-	-/-	-/-
Horse I	127.0	5.2	96.0	2.3	0.45	0.7	6	3318	5.0
Horse J	-/-	-/-	-/-	-/-	-/-	-/-	-/-	1407	2.3
Horse K	-/-	-/-	-/-	-/-	-/-	-/-	-/-	2344	4.4
Horse L	-/-	-/-	-/-	-/-	-/-	-/-	-/-	3713	3.3
Horse M	138.0	3.1	101	2.1	0.59	1.0	6	-/-	-/-
Horse N	138.0	3.3	105.0	2.5	0.61	0.7	7	-/-	-/-
Horse O	134.0	2.8	96.0	2.4	0.59	0.7	9	176	1.3
Horse P	137.0	3.6	104.0	2.5	0.57	0.6	7	1796	-/-
Horse Q	133.0	3.0	92.0	2.5	0.47	1.0	7	3755	3.8
Horse R	-/-	-/-	-/-	-/-	-/-	-/-	-/-	7152	-/-
Horse S	126.0	4.5	86.0	2.1	0.58	1.3	13	14152	6.5
Horse T	132.0	3.0	97.0	2.4	0.65	0.7	12	467	1.7
