# Supplementary material for: Preliminary Investigation of Side Effects of Polymyxin B Administration in Hospitalized Horses
Source: Antibiotics (Basel). 2023 May 5;12(5):854. doi: 10.3390/antibiotics12050854 (PMC10215903; doi:10.3390/antibiotics12050854)
Supplement: Supplementary file 1 [file antibiotics-12-00854-s001.zip › Supplementary 3_ Ataxia gradings of individual horses.pdf]

Supplementary 3

Median daily ataxia scores of 20 hospitalized horses receiving PolyB treatment (6000IU/kg IV q12h) over time. Presented are median scores of three blinded observers gradings on video exams. Horses received 1-3 days of PolyB treatment and neurological examination was performed daily during and three days after the last PolyB dose (post 1-3). Abbreviations: N.a. not assessed

	Day 0	Day 1	Day 2	Day 3	Post 1	Post 2	Post 3
Horse A	n.a.	1	---------	---------	2	1	1
Horse B	n.a.	3	---------	---------	3	1	1
Horse C	n.a.	1	---------	---------	2	0	0
Horse D	n.a.	2	---------	---------	n.a.	2	3
Horse E	n.a.	2	---------	---------	n.a.	n.a.	n.a.
Horse F	n.a.	n.a.	---------	---------	2	2	0
Horse G	n.a.	n.a.	---------	---------	3	0	1
Horse H	n.a.	n.a.	---------	---------	3	n.a.	n.a.
Horse I	n.a.	2	---------	---------	2	2	1
Horse J	n.a.	2	---------	---------	3	3	1
Horse K	n.a.	2	---------	---------	3	3	2
Horse L	n.a.	3	2	---------	2	2	1
Horse M	n.a.	2	2	---------	3	1	1
Horse N	n.a.	n.a.	3	---------	3	2	2
Horse O	n.a.	2	3	---------	2	1	1
Horse P	n.a.	n.a.	3	---------	2	2	1
Horse Q	n.a.	3	3	---------	2	2	1
Horse R	1	1	1	1	---------	---------	---------
Horse S	n.a.	n.a.	2	2	1	2	0
Horse T	n.a.	n.a.	n.a.	3	1	0	n.a.
Median (range)	1	2 (1-3)	2.5 (1-3)	2 (1-3)	2 (1-3)	2 (0-3)	1 (0-3)
